# Critical aspects and challenges for intervertebral disc repair and regeneration—Harnessing advances in tissue engineering

**DOI:** 10.1002/jsp2.1029

**Published:** 2018-07-30

**Authors:** Conor T. Buckley, Judith A. Hoyland, Kengo Fujii, Abhay Pandit, James C. Iatridis, Sibylle Grad

**Affiliations:** ^1^ Trinity Centre for Bioengineering, Trinity Biomedical Sciences Institute Trinity College Dublin, The University of Dublin Dublin Ireland; ^2^ School of Engineering, Trinity College Dublin The University of Dublin Dublin Ireland; ^3^ Advanced Materials and Bioengineering Research (AMBER) Centre Royal College of Surgeons in Ireland & Trinity College Dublin, The University of Dublin Dublin Ireland; ^4^ Division of Cell Matrix Biology and Regenerative Medicine University of Manchester Manchester UK; ^5^ NIHR Manchester Musculoskeletal Biomedical Research Unit, Central Manchester Foundation Trust Manchester Academic Health Science Centre Manchester UK; ^6^ Leni & Peter W. May Department of Orthopaedics Icahn School of Medicine at Mount Sinai New York New York USA; ^7^ Department of Orthopaedic Surgery University of Tsukuba Tsukuba Japan; ^8^ Centre for Research in Medical Devices (CÚRAM) National University of Ireland Galway Ireland; ^9^ AO Research Institute Davos Davos Switzerland

**Keywords:** biomaterials, clinical translation, mechanical compatibility, microenvironment, mimicry, tissue engineering

## Abstract

Low back pain represents the highest burden of musculoskeletal diseases worldwide and intervertebral disc degeneration is frequently associated with this painful condition. Even though it remains challenging to clearly recognize generators of discogenic pain, tissue regeneration has been accepted as an effective treatment option with significant potential. Tissue engineering and regenerative medicine offer a plethora of exploratory pathways for functional repair or prevention of tissue breakdown. However, the intervertebral disc has extraordinary biological and mechanical demands that must be met to assure sustained success. This concise perspective review highlights the role of the disc microenvironment, mechanical and clinical design considerations, function vs mimicry in biomaterial‐based and cell engineering strategies, and potential constraints for clinical translation of regenerative therapies for the intervertebral disc.

## 1 INTRODUCTION

**Figure 1:**
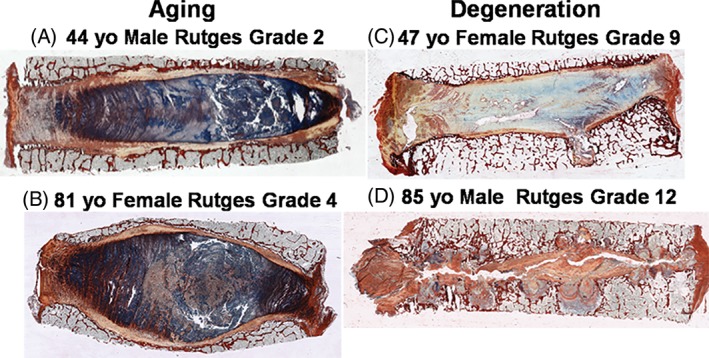
Variations in intervertebral disc (IVD) structure and composition with aging vs degeneration. Picrosirius red/alcian blue (PR/AB) staining of mid‐sagittal sections of four different human IVDs. PR/AB highlights the differences between IVD aging and IVD degeneration. Column 1. Aging: Aging IVDs show subtle changes in structure and composition with retention of overall annulus fibrosus (AF) structural integrity. (A) Forty‐four‐year‐old male IVD retains healthy end plates with only slight irregularities, well‐organized annular morphology, nearly normal nuclear tissue with only slight disorganization, and intense matrix staining. (B) Eighty‐one‐year‐old female IVD shows only slight irregularities in the endplate. It maintains a well‐organized annulus with only slight loss of annular‐nuclear demarcation, and mild loss of nuclear staining intensity. This aged specimen also shows rounded end plates due to osteoporotic changes in underlying trabecular bone. Column 2. Degeneration: Degenerated IVDs show larger changes in structure that disrupt the gross integrity of the AF, the nucleus pulposus, and/or the end plates and changes in composition with loss of staining intensity. (C) Forty‐seven‐year‐old female exhibits multiple irregularities in the endplate including thinning and focal breaks, a loss of boundary demarcation between the nucleus and annulus, and disorganized/fibrotic nuclear matrix and little AB staining. The IVD also displays horizontal fissures that extend into the annulus and disrupt its structure. (D) Eighty‐five‐year‐old male IVD shows severe irregularities in the endplate, disorganization of the nucleus and complete rupture of the annulus. The faint staining shows nearly complete loss of matrix material, leading to collapse of the disc, bulging of the annulus, and areas of bone to bone contact. In this extreme case, there is complete loss of structural integrity of the IVD

Low back and neck pain is associated with the highest burden of musculoskeletal disorders and is a leading cause of global disability with tremendous social and economic impact.^1^, ^2^ It remains clear that the efficacy of operative and nonoperative treatment requires patients with specific indications and precise diagnosis.^3^, ^4^, ^5^ However, precision diagnosis is commonly lacking for patients with discogenic back pain and multiple spinal disorders which can have complex definitions and interacting structural, biological, and inflammatory sources of pain.^6^, ^7^, ^8^, ^9^ Biochemical, cellular, and structural changes in the intervertebral disc (IVD) accumulate over decades. Degeneration‐related structural changes are more prominent than age‐related changes (Figure 1). Certain structural changes with degeneration can directly result in pain and include endplate and annulus‐driven phenotypes while aging changes are often more subtle and not tied to pain.^10^ However, it has long been known that nonpainful control subjects also exhibit structural defects on radiological investigation making it difficult to identify specific structural defects as a pain generator in many patients. From 2008 to 2014, there were substantial increases in the diagnosis of patients with lumbar (33% increase) and cervical (42% increase) spinal disorders in the Medicare database; however, there were also decreases in both lumbar and cervical surgical and nonoperative treatments.^11^ Discordance between diagnosis and treatment trends in the elderly points to a strong need to develop and optimize treatments for spinal care, particularly for the elderly. The burden of back pain affects both young and old patients, highlighting a demand for novel treatment strategies that reduce pain and improve quality of life for all back pain sufferers.

Tissue engineering and regenerative medicine strategies have the potential to address axial back pain and herniation. The complexities of diagnosis and patient selection in back and neck pain conditions highlight a strong need to develop safe and minimally invasive treatments that can repair IVDs and/or prevent painful conditions. In the case of axial back pain, the challenges of identifying a specific source of pain highlights a need for a safe and injectable treatment. As injectable treatments are developed, however, these strategies must also minimize the annulus fibrosus (AF) damage and comorbidities known to occur from IVD puncture, injection, and discography.^7^, ^12^ Current strategies for axial back pain are conservative treatment, physical therapy and oral analgesics which have limited efficacy for many patients. IVD herniation is a specific cause of back and leg pain and disability where discectomy procedures have improved outcomes compared to nonoperative controls.^5^ Discectomy is an effective treatment for IVD herniation, yet even in the case of successful herniation procedures, long‐term complications can include reherniation and recurrent back pain.^13^ Tissue engineering and regenerative medicine treatments offer tremendous potential to repair and regenerate IVD tissues and potentially alter the course from painful to nonpainful conditions for axial back pain and herniation patients. Treating these varied conditions requires development of biomaterials for AF repair, and nucleus pulposus (NP) repair and regeneration. For final repair and regeneration strategies, the IVD tissue has tremendous biological and mechanical demands which must be addressed to achieve successful outcomes. Varying diagnoses also necessitate the development of multiple repair and regeneration strategies that focus on function and mimicry. This narrative review has four objectives, namely to describe: (1) the role of the host/disc microenvironment, (2) mechanical and clinical design constraints, (3) biomaterials and cell engineering for function vs mimicry in the IVD, and (4) challenges of clinical translation for these regenerative repair strategies.

## 2 THE ROLE OF THE DISC MICROENVIRONMENT

Degeneration of the IVD occurs over many years and is influenced to an extent by genetic, environmental and physicochemical effects. However, for normal cellular function and successful tissue regeneration, the local physicochemical microenvironment that is experienced by implanted cells is critical. The degenerated microenvironment of the human IVD is characterized by altered oxygen,^14^ reduced glucose,^15^, ^16^ increased matrix acidity^14^ and elevated levels of proinflammatory cytokines^17^ thus presenting a challenging microenvironment for normal cell function. As the IVD is avascular, the surrounding blood vessels in the cartilage end plates (CEPs) and vertebral bodies supply vital nutrients to the disc primarily through diffusion.^16^ Balance between nutrient transport and cellular consumption rates establishes a concentration gradient throughout the disc of these nutrients and metabolites which in turn markedly affect viability, proliferation and function of cells, and collectively will undoubtedly impact the degree of any subsequent regeneration.

Oxygen levels have been shown to vary considerably in human lumbar and thoracic discs, and do not appear to correlate with age, pathology or stage of degeneration. Concentrations decrease from the AF across the disc structure (19.5%‐0.65%) with average physioxic concentrations in the central region of the NP of between 5% and 10%.^14^ These gradient concentration profiles are dependent on the rate of oxygen transport through the CEP, cellular density and consumption rates. It is well established that cell viability of NP cells is diminished with low glucose but not low oxygen, highlighting the importance of glucose as a limiting nutrient for survival of disc cells. Computational models have predicted a decrease in glucose concentrations from ~5 mM at the disc boundaries to ~0.8 mM in the center of healthy discs (uncalcified),^18^ which can fall below critical levels with increasing calcification and as a function of static strain conditions.^16^ Importantly, cell death has been shown to occur when subjected to glucose concentrations below 0.5 mM for more than 3 days^19^ and in scoliotic discs, low cell viability was found to correlate with low glucose concentrations.^20^

Another important factor is the pH microenvironment due to local lactic acid concentrations (typical range of 2‐6 mM) as a result of glycolysis.^14^ In vivo measurements reveal that pH varies from 5.7 to 7.5 (median, 7)^21^ and can significantly influence cell survival, adversely affect matrix synthesis rates^19^, ^22^ and increase expression of proinflammatory cytokines and pain‐related factors.^23^ Importantly, energy metabolism rates are nonlinear coupled reactions and dependent on the local nutrient and acidic microenvironment.^24^ Equally relevant from a regenerative medicine or tissue engineering perspective, oxygen concentrations appear to play a key role in regulating the phenotype and biosynthetic activity of cells intended for therapeutic applications,^25^ while low glucose concentrations and low pH levels have been found to impair the survival and biological behavior of stem cells.^26^, ^27^, ^28^

In addition, the IVD is subjected to biophysical forces in vivo, such as deformational strain and hydrostatic pressure. In vivo pressures appear to be task‐dependent and vary significantly. Using micro‐pressure transducers, it has been shown that pressures in the IVD (L4‐L5 disc) range from 0.1 MPa when lying prone to 0.95 MPa during jogging to as high as 2.3 MPa when lifting a 20 kg object.^29^ Furthermore, due to the presence of negatively charged proteoglycans, the IVD is an osmotic system, which has been shown to be a potent regulator of gene expression^30^ and matrix synthesis by IVD cells.^31^ Due to diurnal changes alone, the osmolarity can range from 450 to 550 mOsm,^32^ which can also affect subsequent cellular response to biophysical stimulation.^33^

A compounding issue occurs during aging; the CEPs become less permeable due to endplate calcification, which impedes the diffusion and nutrient exchange between the vertebral marrow and the disc itself.^34^ Previous work has shown that occlusion of endplate openings correlates significantly with disc degeneration and is strongest for the endplate adjacent to the nuclear region, suggesting that endplate calcification may impair nutrient transport thereby leading to disc degeneration.^35^ Marrow contact channel surface has been shown to be highest in the center of vertebral endplates compared to peripheral zones near the AF and strongly correlates with effective permeability measurements.^36^ Alterations in mechanical stimuli have also been shown to alter the vascularization and the convective properties of the CEP,^36^ highlighting the role mechanobiological factors may have in triggering CEP changes. In addition, endplate damage or alterations can result in increased communication between the bone marrow and the disc regions. Recent work characterizing the molecular and cellular features of Modic Changes between bone marrow and adjacent discs suggests a proinflammatory and fibrogenic coupling, most likely due to increased biologic communication or “cross‐talk” between the two compartments.^37^

Recent work has also demonstrated that calcium (Ca^2+^) content is consistently higher in human CEP tissue and correlates with grade of disc degeneration. Experiments have shown that increasing levels of Ca^2+^ results in decreases in the accumulation of collagens type I, II, and proteoglycan in cultured human CEP cells through activation of extracellular calcium‐sensing receptors. It is hypothesized that altered or accelerated bone turnover, possibly due to development of osteoporosis, may be responsible for these elevations in calcium levels thereby promoting endplate calcification, impacting tissue permeability^38^ and impeding nutrient transport.

Another important facet is the inflammatory milieu present in degenerated discs. Whether cytokines play a significant role in the initial pathology or if their production is stimulated by the altered physicochemical microenvironment has not been fully elucidated. However, it is evident that inflammatory cascades are potentiated in disc disease and that a multitude of cytokines and inflammatory molecules are involved in these processes which influence cell survival, differentiation and function. Among these, interleukin 1 (IL‐1) and tumor necrosis factor‐alpha (TNF‐α) have received the greatest attention.^39^

Evidence suggests the release of factors from degenerating discs such as nerve growth factor (NGF), brain‐derived neurotrophic factor (BDNF), inflammatory and nociceptive factors also play a vital role in the cross‐talk responsible for activation and recruitment of immune cells as well as promoting neo‐innervation.^9^, ^40^ Additionally, low pH can stimulate the production of both NGF and BDNF^23^ and has been implicated in pain perception.^41^ Therefore, inflammatory cytokines, through their precipitation of neurotrophines, may indeed act as noxious stimulation which sensitizes nerves, and/or initiates in‐growth of nerve fibers into the degenerate disc, thereby exacerbating back pain.^23^

Minimally invasive delivery of cells into the disc space to regenerate matrix, and/or to positively alter the microenvironment and restore functionality may hold significant promise for disc regeneration. Significant advances have been made in identifying potential cell sources and biomaterials for translation, understanding of cellular crosstalk, assessing microenvironmental effects, harnessing developmental biology processes, inflammatory pathways and cascades, and establishment of better preclinical models. However, there remain many unanswered questions for successful translation. These include understanding or identifying: (1) if transplanted cells can survive and sustain the compromised physicochemical microenvironment in vivo; (2) how the delivery of exogenous cells may exacerbate the imbalanced nutrient‐metabolite milieu that exists in degeneration; (3) if functional repair (through the stimulation of neo‐matrix) will result in pain relief; (4) what is the desired composition or quality of matrix required, and if it will be sufficient to sustain the typical biochemical microenvironment or biophysical loads experienced; (5) how to design patient‐specific or personalized therapies to suit unique microenvironments and how such microenvironments can be identified/characterized. Importantly, as the field advances towards more extensive clinical trials for assessing cell‐based therapeutics, there is a clear need to identify specific and suitable cohorts of patients to maximize success. Noninvasive characterization of the biochemical state of the IVD could help to determine or predict if the disc microenvironment is compatible or permissive for cell‐based therapies.

For noninvasive characterization, the gold standard to date has been the Pfirrmann disc degeneration grading system based on signal intensity from T2‐weighted magnetic resonance imaging (MRI) to estimate water content with morphological parameters.^8^ While the Pfirrmann grading scale can classify disc degeneration from Grade I to V, it is primarily based on disc structure rather than the biochemical microenvironment. Enhanced imaging modalities and identification of key biomarkers at an earlier stage are required to deliver improved regenerative outcomes for disc regeneration. Recent studies have emerged utilizing the many facets of MRI to garner information on the biochemical state of the degenerated microenvironment. Quantitative MRI using the relaxation times T1 and T2, the magnetization transfer ratio, and the apparent diffusion coefficient facilitates noninvasive assessment and diagnosis of changes including disc matrix composition (water, proteoglycan, and collagen), integrity (percent collagen denaturation),^42^ and biomechanics which have been shown to correlate strongly with disc tissue degeneration.^43^ Axial T1ρ MRI has been utilized to quantify proteoglycan concentrations which can be related to Pfirrmann grading,^44^ while chemical exchange saturation transfer (CEST) MRI has been employed for in vivo IVD pH level‐dependent imaging without the need for exogenous contrast agents.^45^ T2 mapping has been observed to be particularly sensitive to early and intermediate stage biochemical and mechanical degenerative changes compared with T1ρ in an ex vivo chymopapain digestion lapine model, while both parameters appear to be sensitive to advanced degenerative changes.^46^ Profiling of circulating cytokines or biomarkers may also aid in diagnosing patients with degenerative disc disease with recent work showing that serum levels of IL‐6 were significantly higher in subjects with back pain compared with control subjects.^47^

**Figure 2:**
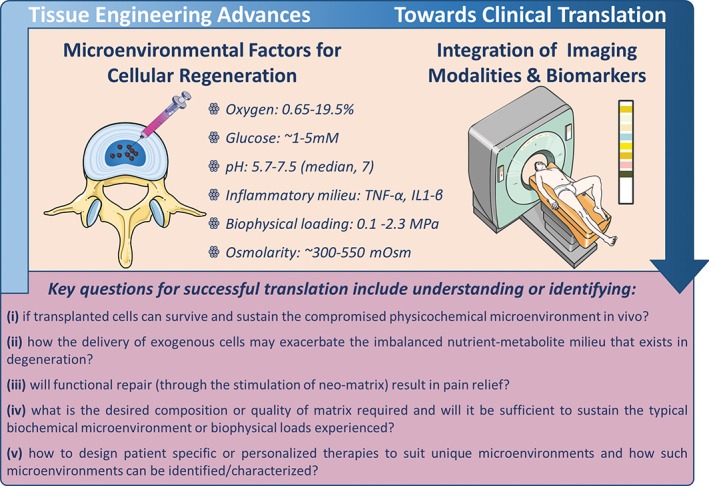
Summary of physicochemical microenvironmental factors and key questions for successful clinical translation. Integrating biomedical imaging strategies with biomarker screening are key aspects to help identify and stratify suitable patient cohorts for cell‐based regeneration

In summary, significant advances have been made in understanding the role of disc microenvironmental factors and their effect on cell viability and function. More sophisticated and integrated diagnostic methods are required to identify and stratify suitable patient cohorts that will benefit from cell‐based therapies (Figure 2). Advances in imaging modalities to quantify the local structural and biochemical microenvironment that is amenable to repair procedures, coupled with biomarkers for tracking repair are obvious targets to address and may provide a suitable strategy and assist in developing personalized and effective treatments for disc degeneration.

## 3 THE IMPORTANCE OF MECHANICAL COMPATIBILITY

As mentioned earlier, IVD herniation is a common cause of back and leg pain, and disability where discectomy procedures have improved outcomes as compared to nonoperative controls.^5^ In the case of successful herniation procedures, herniated IVD tissues are removed to reduce the neuropathy condition. However, the IVD remains unrepaired with risk of long‐term complications including reherniation and recurrent back pain,^13^ whereby reherniation rates after discectomy are reported to be 5% to 25%.^13^, ^48^ Selection of how much tissue to remove can be challenging, where limited discectomy (with relatively little tissue removal) can result in increased reherniation rates, while aggressive discectomy can decrease the risk of reherniation but worsen overall outcomes.^49^ Importantly, the clinical challenge of reherniation risk has slowed the translation of tissue‐engineered strategies for the treatment of human spinal pathologies, as tissue‐engineered biomaterials and other implants run the risk of reherniation, which results in neuropathy and increased disability and pain. Indeed, reherniation risk is a single biomechanical design constraint for implanting tissue engineering and regenerative medicine treatment solutions. Design criteria for biomaterials for IVD repair include biocompatibility, biomechanical design criteria, and clinical applicability criteria. Mechanical design criteria include those criteria that are most likely to achieve mechanical compatibility to promote longevity and reduce reherniation risk. Clinical applicability criteria are those required to enable and facilitate clinical translation within the constraints of the clinical environment and patient safety.

Implants for IVD repair can improve spinal health and reduce painful conditions by primarily restoring IVD height and biomechanical properties to the healthy condition with negligible risk of herniation. Pain relief and long‐term performance/efficacy can likely be further enhanced if these implants are functionalized to deliver drugs, biological factors or cells. As such, the development of biomaterials capable of achieving such design goals remains an active area of investigation with several biomaterials options.^7^, ^50^, ^51^, ^52^ Design considerations remain constant for all biomaterial choices to achieve the biomechanical demands on the spine. Negligible or low herniation risk is a critical design requirement that can be addressed with a biomaterial that strongly adheres to the native tissue, but this goal remains a research challenge and active area of investigation. However, suturing and other AF closure devices also offer the potential to reduce reherniation risk^53^ alone or in combination with additional biomaterial delivery. A space‐filling material with capacity to restore IVD height commonly requires an amorphous biomaterial capable of being injected into the IVD space. Herniation can also occur due to mismatch of biomaterials with native tissues that can overpower even with the strongest annular closure devices with the rigors of fatigue over extensive loading cycles.

**Figure 3:**
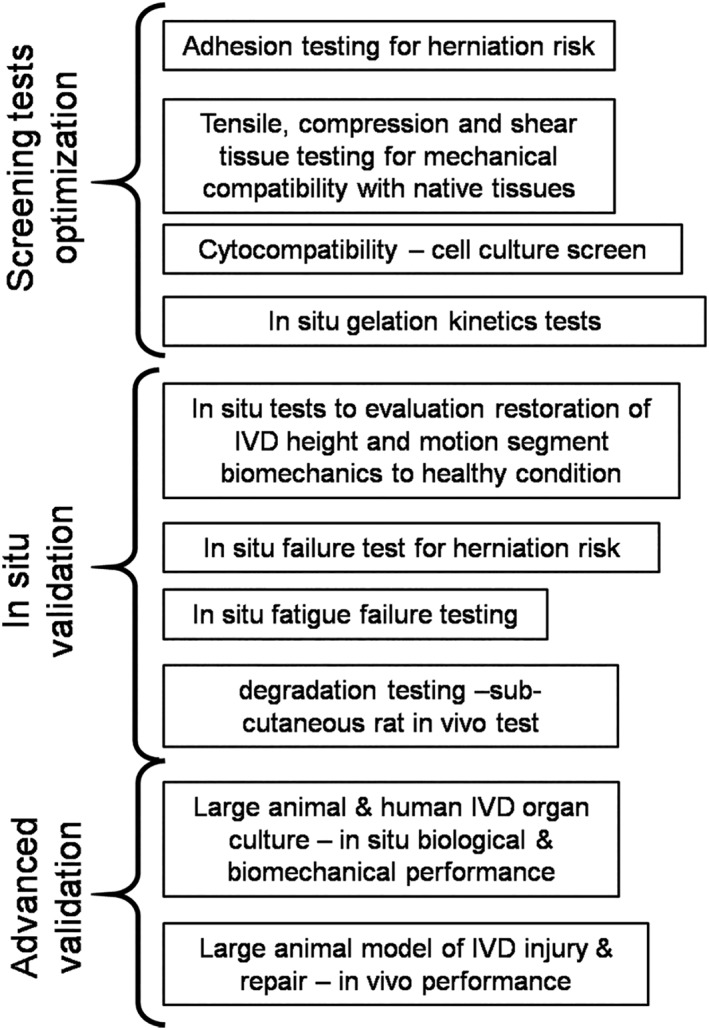
Testing paradigm for evaluating intervertebral disc (IVD) repair strategies. Screening tests involve high throughput evaluations that can rapidly assess materials. Testing process progresses to in vivo validation. This figure was modified from Long et al^51^

A consistent set of evaluation criteria for IVD repair biomaterials can facilitate comparisons of varying biomaterials from different laboratories and more rapidly advance the field. A testing paradigm for AF repair biomaterials has been suggested that spans from rapid screening tests for optimization, in situ validation tests, and advanced validation tests that is modified for more general use for IVD repair (Figure 3). Screening tests for optimization are designed to evaluate priority parameters to rapidly assess if the biomaterial will meet required design parameters. Importantly, they are intended to be adaptable for high throughput testing and include adhesion testing, material property determination and cytocompatibility assessments.^51^ To date, many of these optimization tests are performed on isolated tissue samples with more free‐boundaries than would be found in situ which creates high shear stresses.^54^ As a result, the adhesion strength and material property parameters obtained from a screening test are commonly best used as a relative comparison rather than an absolute measurement value. Gelation kinetics tests (eg, rheometer measuring shear modulus through time) can evaluate if the material will solidify rapidly enough to be consistent with current medical procedures. In situ gelation kinetics must also assess if the biomaterial is capable of solidifying and/or being implanted in situ, since gelation conditions in the human IVD clinical condition can vary substantially from those of the lab.

**Figure 4:**
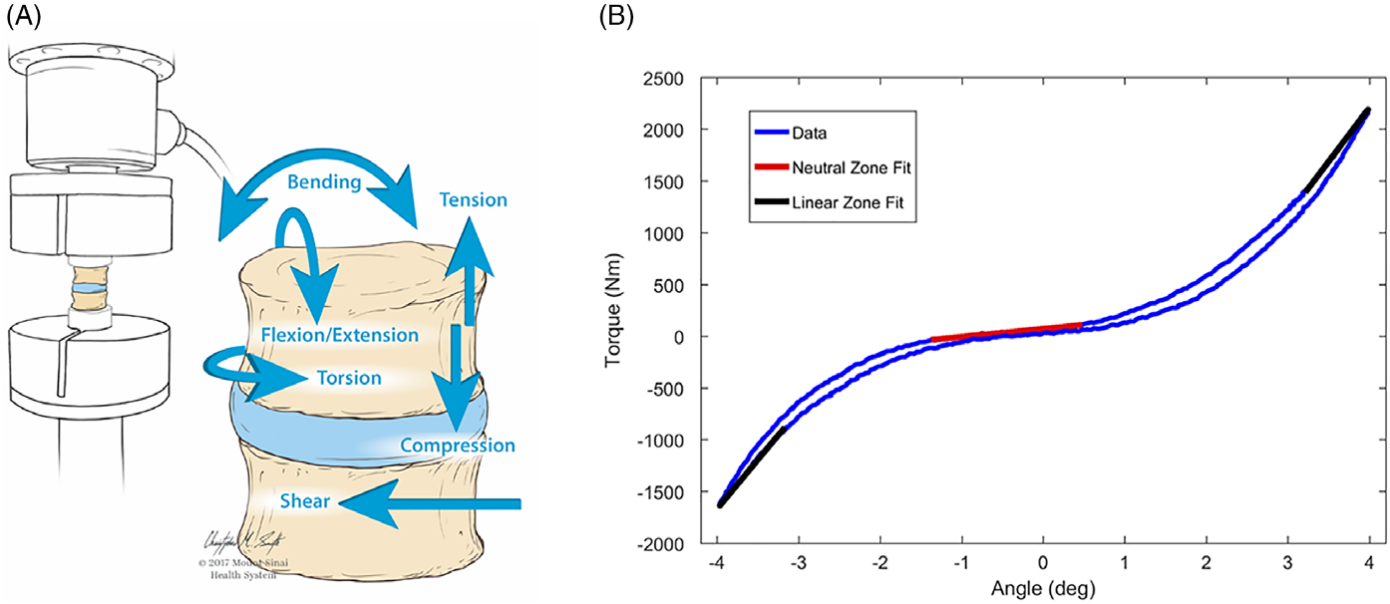
In situ biomechanical testing for advanced screening can include six degrees of freedom testing to evaluate spine biomechanical properties. These biomechanical properties can characterize the neutral zone as well as the stiffness and hysteresis. In situ biomechanical validation tests can also include acute and fatigue failure simulation

In situ tests provide validation and failure testing that is more akin to the in vivo condition, since they retain many important features of the spinal column found in vivo (Figure 4). Such in situ biomechanical tests can determine if biomechanical behaviors can be restored to the healthy condition following simulated injury or degeneration and can also be used to evaluate failure loads and fatigue failure. In situ failure tests load IVDs mechanically until failure to determine if the material has high herniation risk,^55^ while in situ biomechanical tests evaluate restoration of IVD mechanical properties following creation of a defect.^51^ In situ failure tests can also include more rigorous fatigue loading to evaluate implant failure. Since multiaxial loading over many cycles results in IVD damage accumulation and is a likely cause for AF damage and herniation, it is accepted that multiaxial testing is an important in situ loading condition for implant safety. Wilke et al developed elegant multiaxial testing procedures to assess implant failure under extreme and fatigue loading conditions.^56^ In vivo degradation tests involving subcutaneous and/or in situ implantation of a hydrogel in a small animal model can assess the in vivo degradation rate and inflammatory response. Advanced validation tests are the most involved tests using living organ culture systems and/or animals. Organ cultures can characterize healing potential, degradation and mechanical behaviors of repair strategies using human IVDs and/or large animal IVDs (reducing the need or number of whole animals required)^57^, ^58^ but have limits since they lack the immune system of a live animal. Large animal models are important for advanced validation tests since measurements can include assessments of in vivo healing, biocompatibility, biomechanical restoration and in some cases behavioral measurements predictive of painful responses.^59^, ^60^

## 4 FUNCTION VS MIMICS: BIOMATERIALS AND CELL ENGINEERING

**Figure 5:**
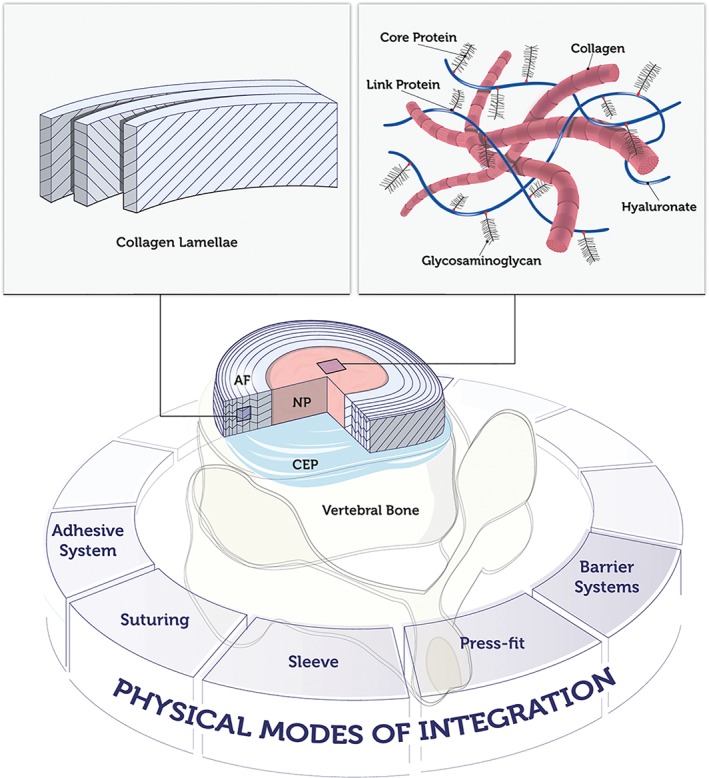
Nucleus pulposus (NP) mimics may consist of natural or synthetic hydrogels, decellularized matrix, specific adhesion proteins or osmo‐responsive molecules; while annulus fibrosus (AF) mimics may be realized with crosslinked hydrogels or fibers arranged in oriented angle‐ply laminates. Challenges in reproducing the authentic tissue include: interfaces NP‐AF‐cartilaginous endplate (CEP)‐vertebrae; integration with native structures; degradation and remodeling properties; regulation of osmotic pressure; complexity of natural matrix, glyco‐pattern, small molecules; cell adhesion properties; cell phenotype regulation; in vitro‐ex vivo‐in vivo translation. Potential strategies for implant integration are displayed

The IVD is a complex organ consisting of interrelated tissues that differ considerably in structure and function. In general, three types of tissues are distinguished, namely the NP, AF and CEP, while the transition zones and interfaces between these tissues play a fundamental role for their interaction and integration. Either prolonged detrimental impact or an acute traumatic event can disturb the anabolic‐catabolic balance towards tissue breakdown and loss of function. Current clinical treatments are often not satisfactory in the long‐term and can even trigger a degenerative cascade in adjacent IVDs.^61^ It is therefore hypothesized that effective and sustained regeneration may be achieved by therapies that aim to closely mimic the composition and structure of the native tissues. A multitude of structural interventions using biomaterials with or without functional units have been designed for NP, AF or whole IVD with the intent to reproduce the natural conditions; whereby both substitutive and regenerative strategies have been pursued.^50^, ^62^, ^63^, ^64^ Nevertheless, it remains challenging to develop functional tissues ex vivo, knowing that the native situation can only be approximately simulated (Figure 5). From a practical perspective, injectable, void filling, in situ reacting, minimally invasive treatments are most desirable and may offer the greatest potential for clinical translation. Meanwhile, the required level of complexity of an effective tissue‐engineered biomaterial is still a matter of debate.^7^, ^65^, ^66^

Physiological NP matrix contains 70% to 90% water, while its dry weight consists of 20% collagen, mainly type II, and 30% to 50% proteoglycan.^67^ For NP regeneration, it is crucial to address the water content since tissue dehydration is a major hallmark of the degenerative cascade. Hydrophilic materials such as hydrogels have widely been investigated to compensate for the water uptake properties of the NP. Injectable hydrogels represent an attractive minimally invasive approach. They can also be combined with cells to establish and maintain tissue homeostasis in cases where endogenous cells are deficient, abnormally functioning or inactive.^68^ Natural‐origin hydrogels can be processed but do not need to be synthesized, which reduces production costs. Furthermore, they are generally cytocompatible, bioactive, and participate in the physiological turnover process.^69^ Of note, the IVD matrix metabolism is extremely slow, so the degradation rate of an ideal material should be compatible with the natural processes. Despite the extensive literature on the use of natural hydrogels for NP therapy, the range of core materials appears restricted. Most often described hydrogels include hyaluronic acid, collagen type I or II, fibrin, gelatin, alginate, chitosan, and gellan gum.^50^, ^68^ Synthetic hydrogels offer a valid alternative, as they can be produced in a standardized and reproducible way and can be tuned towards the desired mechanical and degradation properties. Examples of hydrogels described for NP tissue engineering applications include polyethylene glycol, polyvinyl alcohol, polyvinyl‐pyrrolidone, polyurethane, and cellulosic.^50^, ^68^, ^70^ Another molecular approach involves the design of injectable sulfonate‐containing hydrogels with high fixed charge density and swelling pressure, which may serve as biomimetic glycosaminoglycan analogs.^71^ Such materials that swell in situ to form a nondegradable gel as NP replacement aim at restoring disc height, though the cellular response and long‐term tissue integration are also essential arguments to be considered.^72^ While pure components are typically characterized by a low risk of cytotoxicity, their swelling behavior, durability, and the host tissue reaction need to be carefully considered to prevent subsidence or extreme overload, potentially causing CEP/EP fracture.^73^, ^74^

Mimicking the natural NP characteristics is essential for both immediate tissue restoration and cellular activation. The phenotype of the cells embedded in or exposed to a material is highly influenced by its physical, chemical, and mechanical quality. Despite the prevalence of type II collagen, type I collagen has more often been used due to the remarkably lower cost and superior availability. A crosslinked formulation of type II collagen showed improved stability compared to the noncrosslinked molecule with still favorable cell differentiation response.^75^ Atelocollagen is a low‐immunogenic derivative of collagen that has been successfully used in vivo.^76^, ^77^ Proteoglycans contain abundant polysaccharide chains, making polysaccharide‐based structures such as chitosan, alginate, gellan gum, or carboxymethylcellulose potentially suitable for NP regeneration.^68^, ^70^, ^78^, ^79^, ^80^ Nevertheless, these polymers are not natively present in the NP, and it is uncertain whether the cell and tissue responses, particularly considering the local microenvironment, are appropriate and compatible. Hyaluronan as an important natural component of the NP matrix is a promising material for NP repair. Thermoreversible hyaluronan‐based hydrogel has been shown to promote the phenotype of NP cells and facilitate appropriate differentiation of bone marrow stromal cells in vitro and in organ culture.^81^, ^82^ To more closely approximate the NP matrix, crosslinked atelocollagen type II‐based scaffolds containing varying concentrations of aggrecan and hyaluronan were investigated, although their in vivo effect remains to be explored.^83^ IVD‐specific ECM‐mimics that address the cellular response may prove more sustainable. Recently, a panel of laminin mimetic peptides conjugated to polyacrylamide gels were reported to promote an immature healthy NP phenotype after culture on soft peptide gels.^84^ The results demonstrated that cell‐matrix interactions play a crucial role in gaining and maintaining a regenerative phenotype and activity; thus, mimicking the ECM structure alone may not be sufficient without mimicking its functional cellular microenvironment. An attractive approach consists in the generation of decellularized matrices from healthy NP. Decellularization processes obtain biomaterials that represent the native tissue microstructure and biochemistry, supporting cellular adaptation.^85^, ^86^ Further preclinical studies are warranted to compare such matrices with other synthetic or natural biomaterials. Recently reported studies are only at the beginning of identifying the molecular patterns that determine the NP cell phenotype.^87^, ^88^, ^89^

The AF is a multilamellar structure composed of 70% collagen, primarily type I, and 10% proteoglycan in dry weight.^67^ One of the challenges of AF tissue engineering is the gradual transformation of structure and biochemistry from the outer AF to the inner AF and the NP that cannot easily be reproduced ex vivo. Various biomaterials have been suggested as a basis for AF repair, including collagen, atelocollagen, silk fibroin, poly‐lactic‐co‐glycolic acid, and poly‐caprolactone (PCL).^7^, ^68^ Electrospun fibers generated from PCL are highly anisotropic and closely replicate the AF structural hierarchy; when seeded with mesenchymal stromal cells, these scaffolds promoted the deposition of an organized collagen‐rich ECM that approached the angle‐ply multilamellar architecture of native AF.^52^ Porous silk scaffolds and their derivatives have also shown promising characteristics for AF tissue engineering, supporting AF‐like matrix production of seeded cells.^90^ Atelocollagen scaffolds seeded with autologous AF cells were effective in treating small AF defects in a rabbit in vivo model.^91^ Nevertheless, it is still uncertain whether such hydrogels and fibers maintain the strength and robustness required to integrate with the adjacent AF and bony tissues long term.

Many AF repair materials have been developed as components of whole tissue‐engineered IVDs. For example, cell‐seeded composites consisting of crosslinked bone matrix gelatin acting as the AF and acellular cartilage matrix as the NP component was shown to promote the development of IVD‐like tissue in an ectopic in vivo model.^92^ An IVD construct based on contracted collagen AF and alginate NP, implanted in a canine cervical total discectomy model, was maintained over several months, although the long‐term functionality of such tissue‐engineered whole IVDs remains a challenge.^93^ The role of an organized AF structure, compared to a scaffold without any lamellar pattern structure, and its importance for the success of the implant is still not well defined. While an oriented lamellar structure may not be required to meet the goal of adhesion or defect filling, the resistance to deformation and tensile circumferential strains clearly depends on the structural organization of the tissue‐engineered implant.^94^ Biphasic scaffolds composed of a collagen‐glycosaminoglycan composite have been fabricated to structurally mimic the NP. The construct consisting of multiple lamellae of crosslinked collagen membranes making up the AF, showed mechanical performance comparable to the native IVD; whereby the constructs containing 10 AF‐like lamellae presented superior properties overall.^95^

Ideally, integration of implants means cellular integration with phenotype maintenance, matrix integration, and function preservation. Nonetheless, further developments are needed that support integration of the tissue‐engineered grafts into the native structures, particularly the cartilaginous or bony endplates. Addition of endplate mimicking components may help to facilitate integration into the vertebral bone and maintain the function of the implant, preventing rapid proteoglycan loss.^96^, ^97^, ^98^ Functionalization of materials with specific ECM components, bioactive factors, or nucleic acids will help with directing the native or therapeutically delivered cells towards the desired phenotype.

### 4.1 Cell engineering

Advances in cell‐engineering technologies have resulted in new emerging approaches that aim to enhance the survival and effectiveness of transplanted cells. The native IVD cell phenotype is likely to be optimally adapted to the challenging microenvironment. Since the proliferation rate and metabolic activity of autologous disc cells are generally poor, ex vivo cell stimulation by coculture with mesenchymal stem cells (MSCs),^99^ growth factors or gene delivery^100^, ^101^ has been proposed to increase the performance of these cells. As an alternative, MSCs derived from bone marrow or adipose tissue have been investigated for IVD regeneration based on their ability to differentiate in response to the microenvironment and on their anti‐inflammatory and immune‐modulatory activity.^102^ While promising outcomes have been reported in preclinical and clinical studies in terms of symptom improvement and disc restoration, the effective cell population and the mechanisms of action are still poorly defined, and the survival rate of MSCs in the IVD environment is still a matter of debate.^103^, ^104^ Ex vivo predifferentiation of MSCs towards an NP cell‐like phenotype prior to implantation might enhance their persistence and effectiveness in the IVD environment. Culture under reduced oxygen conditions, coculture with IVD cells, stimulation with growth and differentiation factor (GDF)‐5, GDF‐6 and/or TGF‐beta have been shown to induce an NP‐like phenotype in human MSCs.^105^, ^106^, ^107^, ^108^ Nevertheless, it remains to be investigated whether ex vivo conditioning of MSCs significantly improves there in vivo performance and which factors are most effective.

Notochordal cells that are present in the human IVD until early childhood but are not detected in the adult have been shown to protect and stimulate the mature NP cells. Several in vitro studies have confirmed the anabolic, antiapoptotic, and antiangiogenic trophic effects of notochordal cell‐derived factors, vesicles and notochordal cell‐derived matrix.^109^, ^110^, ^111^, ^112^, ^113^ Cell engineering technologies generating notochordal cells may thus be explored for emerging cell therapies. Induced pluripotent stem cells (iPSCs), having similar characteristics in terms of pluripotency as embryonic stem cells, offer such possibilities. Liu et al^114^, ^115^ demonstrated that human iPSCs could differentiate into notochordal cell‐like cells, expressing notochordal markers such as Brachyury (T), and cytokeratin‐18, when they were cultured in the presence of porcine NP tissue matrix. Recently, Tang et al^116^ described the differentiation of human iPSCs into NP‐like cells. The authors used a stepwise, directed differentiation towards mesodermal lineage, followed by notochordal lineage, through application of chemically defined medium and growth factor stimulation. Cells adopted a vacuolated NP cell morphology and expressed NP cell surface markers, including CD24, LMα5, and Basp1. These pioneering studies provide important insights into the processes of NP cell maturation and may finally lead to new cell sources for therapeutic purposes. In terms of AF repair, the lack of specific markers for functional AF cells has hampered similar developments. Recently, new markers such as CD146 and Mohawk (MKX) have been identified that characterize the functional AF cell phenotype,^117^, ^118^ opening the door for future cell engineering approaches. Notably these technologies may represent new strategies for unlimited generation of functional IVD‐like cells; though in vivo studies will be needed to assess the therapeutic values of cell engineering developments for IVD repair and regeneration.

## 5 CLINICAL TRANSLATION

Translating IVD repair strategies to humans requires advanced validation methods and clear patient selection criteria. Degeneration‐induced axial back pain requires injectable biomaterials capable of cell delivery, and/or NP repair and regeneration to stop or reverse the degenerative cascade. For herniation patients, the AF must also be repaired since accelerated degeneration after discectomy or conservative therapy for lumbar IVD herniation is well‐established.^13^, ^119^, ^120^ Small and large animal testing is commonly required prior to human clinical translation. Nevertheless, it needs to be considered that all animal models have limitations and generally do not reproduce the mechanisms of human disc degeneration or herniation.^59^, ^121^, ^122^ Animals experiencing spontaneous disc degeneration or herniation such as certain canine breeds may represent attractive models for evaluation of new therapies.^123^ Some devices for AF repair such as Barricaid, NuCore, Neudisc, DiscCell, DASCOR, BioDisc, and NucleoFix have been developed and approved for clinical use; however, none of the currently available devices promote tissue regeneration and their efficacy has yet to be demonstrated fully.^50^ Ideal intraoperative AF and NP repair methods would prevent reherniation, seal the remaining defects, restore biomechanical function, and reduce the likelihood of recurrent pain.^124^

Successful translation of a repair method for clinical application must address patient needs and be adaptable to the constraints of the clinical setting in addition to meeting the biological and biomechanical requirements described. The treatments must be easily delivered via injection, or implantable at the time of procedure when discectomy is being performed. Injectable biological therapy appears ideal for meeting this need following discectomy to fill the gap in the NP and repair AF fissures. Discectomy procedures are relatively short, and the material must remain in place following implantation.^51^ Consequently, gelation time of an injectable gel is an important parameter to define feasibility of clinical translation. The repair also needs to be able to withstand loading immediately when the patient undergoes the procedure and/or recovers from anesthesia and is subjected to dynamic loading associated with normal activities of daily life.

Clinical patient selection is another important consideration. Inclusion criteria for recently reported clinical trials for regenerative cell‐based therapies involved persistent lumbar discogenic low back pain for more than 6 months, and degenerative changes in the IVD (Pfirrmann grade 3 and more, disc height loss over 50%, positive provocative discography).^125^ While chronic low back pain patients can have multiple sources of pain that do not directly come from IVD pathology, it is notable that clinical trials on cell therapy injections into IVDs appear to have potential to improve painful conditions.^126^ Furthermore, IVD repair techniques have potential to repair or reverse degenerative changes in IVDs but would not address degenerative changes in facet joints or other spinal deformity conditions that would likely require augmentation with instrumentation. Therefore, clinical patient selection is likely to be varied and to require multiple repair methods to select the most suitable device/technique for IVD repair. For intraoperative repair methods for lumbar disc herniation patients during discectomy, NP and AF can be repaired with biomaterials as replacement. In this scenario, the purpose and function of the replacement biomaterial will be to prevent or delay the progression of lumbar spine degeneration, to prevent reherniation, and to reduce chronic painful conditions. On the other hand, when the disc repair/regenerative therapy is minimally invasive and to be applied via fluoroscopic injection, for example, the main purpose will be to promote regenerative changes of the IVD with reduced pain, recovery of biomechanical properties and reversal of the degenerative cascade. Injectable therapies have the potential to prevent patients from progression of IVD degeneration in the future and may perhaps be most suitable for patients with early stage disc degeneration. In comparison to more invasive surgery such as intraoperative repair/replacement, the added advantage of injectable delivery is the reduced volume of material; however, there needs to be a benefit over risk of possible progressive degeneration which has been observed after IVD puncture.^7^, ^12^

## 6 CONCLUSIONS

Several biomaterial strategies exist for tissue‐engineered IVD repair, replacement, and regeneration. Successful IVD repair remains an unmet clinical need due to the biological, biomechanical and clinical challenges that the repair biomaterial must face. The unique and harsh biological microenvironment in the IVD limits cell matrix production and often requires a biomaterial to help protect and ensure containment of cells in situ, while promoting viability and maintaining the desired phenotype. Once a biomaterial is injected into the IVD, it risks extrusion and reherniation due to significant mechanical loads that persist in normal daily activities, which could exacerbate the clinical condition, and risk further complications. As a result, biomaterials must undergo robust and rigorous biomechanical testing to ensure biomechanical compatibility and reduce the risk of herniation or fatigue failure. Biomaterials in development need to focus on functional mimicry of the native IVD structure. Tissue‐engineered implants must be compatible with the clinical environment and specifically selected to address the unique clinical condition of the patient. Tissue engineering and regenerative medicine continues to advance at an astounding rate and it is likely that engineered biomaterials and cells will be capable of overcoming the challenging biological, biomechanical and clinical constraints required for IVD repair to improve patient outcomes.
